# The Effectiveness of Antibiotics in Managing Bacterial Infections on Bite Sites following Snakebite Envenomation

**DOI:** 10.3390/toxins15030190

**Published:** 2023-03-03

**Authors:** Subramanian Senthilkumaran, Anika Salim, José R. Almeida, Jarred Williams, Pradeep Vijayakumar, Angayarkanni Thirunavukarasu, Markellos Alexandros Christopoulos, Harry F. Williams, Ponniah Thirumalaikolundusubramanian, Ketan Patel, Sakthivel Vaiyapuri

**Affiliations:** 1 Manian Medical Centre, Erode 638001, Tamil Nadu, India; 2 School of Pharmacy, University of Reading, Reading RG6 6UB, UK; 3 Toxiven Biotech Private Limited, Coimbatore 641042, Tamil Nadu, India; 4 The Tamil Nadu Dr M.G.R Medical University, Chennai 600032, Tamil Nadu, India; 5 School of Biological Sciences, University of Reading, Reading RG6 6UB, UK

**Keywords:** antibiotics, Russell’s viper, *Daboia russelii*, snakebite envenomation, wound infections, antibiotic sensitivity, antibiotic resistance

## Abstract

Snakebite envenomation (SBE) is a life-threatening medical emergency with a high mortality rate. Common secondary complications following SBE, such as wound infections, are significant due to their impact on worsening local tissue damage and causing systemic infection. Antivenoms are not effective to treat wound infections following SBE. Moreover, in several rural clinical settings, broad-spectrum antibiotics are often used without clear guidelines or based on limited laboratory data, resulting in undesirable side effects and exacerbated treatment costs. Therefore, robust antibiotic strategies should be developed to tackle this critical issue. Currently, there is limited information available on the bacterial profiles of SBE-induced infections and antibiotic susceptibility. Hence, it is essential to improve the knowledge of bacterial profiles and their antibiotic sensitivity in SBE victims to develop better treatment strategies. This study aimed to address this issue by examining the bacterial profiles of SBE victims with a specific focus on Russell’s viper envenomation. The most frequently found bacteria in the bites of SBE victims were *Staphylococcus aureus*, *Klebsiella* sp., *Escherichia coli*, and *Pseudomonas aeruginosa*. Linezolid, clindamycin, colistin, meropenem, and amikacin were some of the most effective antibiotics for commonly grown bacteria in SBE victims. Similarly, ciprofloxacin, ampicillin, amoxiclave, cefixime, and tetracyclin were the least effective antibiotics for common bacteria found in the wound swabs of SBE victims. These data provide robust guidance for infection management following SBE and offer useful insights to aid in designing effective treatment protocols for SBE with serious wound infections in rural areas where laboratory facilities may not be readily available.

## 1. Introduction

Snakebite envenomation (SBE) is a serious medical emergency that often results in deaths, disabilities, and socioeconomic consequences in impoverished communities of developing countries [1]. India accounts for a high SBE incidence and mortality (58,000 deaths every year) rate, and this can be attributed to widespread myths/misconceptions about snakes and snakebites as well as inadequate public awareness to seek prompt hospital treatment following bites [2,3]. Russell’s viper (*Daboia russelii*) appears to be the most medically important venomous snake in India, as it causes a majority of hospital admissions, deaths and permanent disabilities [4]. Its venom is rich in phospholipases A_2_ and proteolytic enzymes and causes a wide range of debilitating effects on the body of victims that require timely administration of antivenom and other relevant clinical interventions [5]. Secondary complications arising in patients bitten by Russell’s viper may require additional and complementary therapies, such as antibiotics, haemodialysis, analgesics, and transfusion of plasma/blood cells to achieve a favourable clinical outcome [6,7]. Therefore, it is important to understand the secondary complications induced by SBE to develop better treatment strategies.

Microbial (specifically bacterial) infections are a common secondary consequence of SBE, especially in viper bites, due to the extensive local tissue damage [8,9]. These infections can lead to serious complications such as exacerbating necrosis, systemic infections, and septic shock [10]. The cause of these infections can be diverse, including the snake’s oral microbiome, the environment, and opportunistic bacteria growing on human skin [11]. A recent study found the presence of several drug-resistant bacteria in the oral microbiome of a Russell’s viper and the inefficacy of conventionally used antibiotics such as amoxiclave and penicillin [12]. However, a range of antibiotics are often used in several clinical settings, specifically in rural areas, without appropriate justification to treat SBE-induced bacterial infections, and this frequently results in exacerbated treatment costs and unwarranted side effects. Moreover, the use of antivenom is ineffective in treating SBE-induced microbial infections. These issues highlight the urgent need for further research on this clinically relevant but understudied medical complication associated with SBE. This will pave the way to developing improved and cost-effective clinical management strategies for SBE with robust guidance to tackle a wide range of SBE-induced complications, including infection. In this study, we document the bacterial profiles in local bite sites of 266 SBE victims and provide guidance for the effective management of SBE-induced infections which would be mainly useful in under-resourced clinical settings in rural areas.

## 2. Results

### 2.1. Study Population

**Figure 1:**
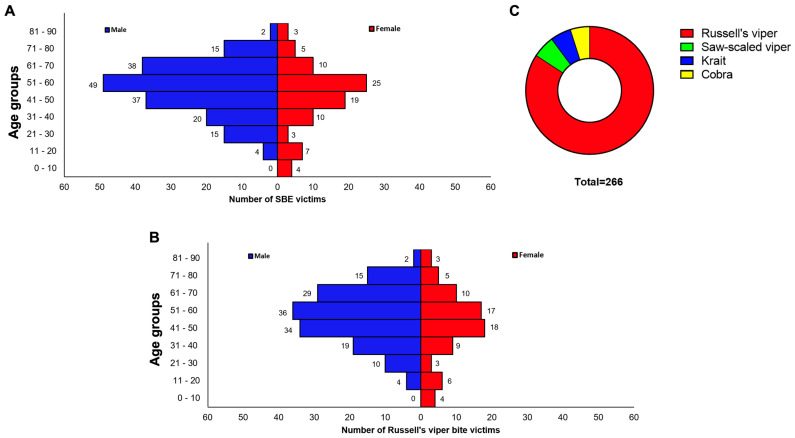
Characteristics of SBE victims included in this study. (**A**) The total number of SBE victims included in this study was organised based on their gender and age groups. (**B**) Different snake species involved in bites of total SBE victims. (**C**) Russell’s viper bite victims were classified based on their gender and age groups.

To determine the bacterial profiles of SBE-induced infections and their antibiotic sensitivity, we recruited SBE victims from January 2021 to December 2022 at Manian Medical Centre, Tamil Nadu following exclusion and inclusion criteria (as detailed in methods). Following an initial screening, a total of 266 SBE victims (180 (67.7%) males; 86 (32.3%) females (Figure 1A)) were included in this study. The patients’ cohort comprised 0 males (0%) and 4 females (1.5%) aged from 0 to 10; 4 males (1.5%) and 7 females (2.6%) aged 11–20, 15 (5.6%) males and 3 (1.1%) females aged 21–30; 20 males (7.5%) and 10 females (3.8%) aged 31–40; 37 males (13.9%) and 19 (7.1%) females aged 41–50; 49 males (18.4%) and 25 (9.4%) females aged 51–60; 38 (14.3%) males and 10 (3.8%) females aged 61–70; 15 males (5.6%) and 5 females (1.9%) aged 71–80; and 2 males (0.8%) and 3 females (1.1%) aged 81–90 years (Figure 1A). The youngest patient included in this study was 8 years old and the oldest patient was 85 years old, with a mean of 50.8 (SD = 16.42) and median of 53 years (IQR: 42 to 62 years). For the Russell’s vipers, the minimum age was 8 years old, and the maximum age was 56, with a mean of 50.7 (SD = 16.9) and a median of 52 (IQR: 41.5 to 63 years).

Notably, this study population included a total of 224 Russell’s viper bite victims (Figure 1B) with 42 victims bitten by other snakes. The distribution of patients across different age groups in Russell’s viper bite victims is similar to the total number of patients (Figure 1C). Due to the high number, we performed a rigorous analysis of bacterial infections from Russell’s viper bite envenomation compared to the others. The 42 patients bitten by other snakes included 13 by cobras (*Naja naja*), 14 by kraits (*Bungarus caeruleus*), and 15 by saw-scaled vipers (*Echis carinatus*).

### 2.2. Diverse Bacterial Strains Were Identified in Wound Swabs from SBE Victims

**Figure 2:**
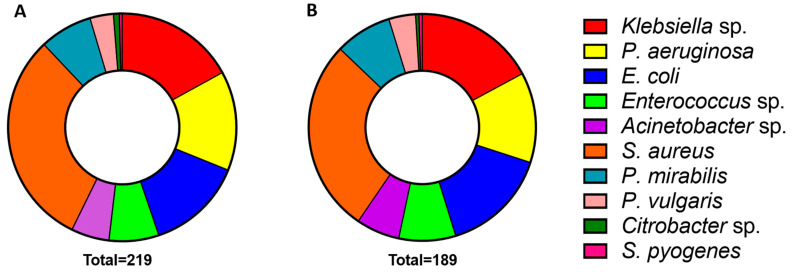
Summary of diverse bacterial strains identified in wound infections of total SBE (**A**) and Russell’s viper bite victims (**B**).

The bacterial strains in wound swabs collected from SBE victims were identified using standard bacterial culture methods in a clinical laboratory associated with the hospital. The bacterial growth was identified in 219 patients; in others, there was no visible growth found. A diverse range of bacteria was identified in these patients (Figure 2A). Most samples displayed a single bacterial growth, whilst 22 patients showed the growth of two different bacteria. These results indicate that *Staphylococcus aureus* was the most observed bacteria as it was found in 74 (31%) patients. This was followed by *Klebsiella* sp. [41 patients (17%)], *Pseudomonas aeruginosa* [34 patients (14%)], and *Escherichia coli* [33 patients (14%)]. All other (six species) bacteria were identified in a small number of patients (Figure 2A). The total bacterial population included three Gram-positive strains (*Enterococcus* sp., *Staphylococcus* *aureus* and *Streptococcus pyogenes*) and four Gram-negative strains (*Acinetobacter* sp., *Citrobacter* sp., *Escherichia coli*, *Klebsiella* sp., *Pseudomonas aeruginosa*, *Proteus mirabilis* and *Proteus vulgaris*). A total of 189 Russell’s viper bites and 30 patients bitten by other snakes displayed bacterial growth. The patients bitten by Russell’s viper displayed a similar pattern of bacterial profiles to the total patient population (Figure 2B). The most common strains present in Russell’s viper patients were *Staphylococcus aureus* (*n* = 58, 31%), *Klebsiella* sp. (*n* = 36, 19%), *Escherichia coli* (*n* = 32, 17%), and *Pseudomonas aeruginosa* (*n* = 27, 14%). Similarly, the least common strains were *Acinetobacter* sp. (*n* = 13; 7%), *Proteus vulgaris* (*n* = 8; 4%), *Citrobacter* sp. (*n* = 1; 1%), and *Streptococcus pyogenes* (*n* = 1; 1%). Of the thirteen patients bitten by cobra (*N. naja*), ten patients presented with *Staphylococcus aureus* and two with *Klebsiella* sp. One patient did not show any bacterial growth. No secondary bacterial strains were found in these patients. Of the patients bitten by kraits (*B. caeruleus*) (*n* = 14), three patients presented with *Staphylococcus aureus*, two with *Klebsiella* sp., and one with *Pseudomonas aeruginosa*. No bacterial growth was found in eight patients. On the other hand, in the analysis of the bacterial data of saw-scaled vipers (*E. carinatus*) bite patients (*n* = 15), the following bacteria were identified: *Pseudomonas aeruginosa* (6), *Staphylococcus aureus* (3), *Proteus mirabilis* (1), *Escherichia coli* (1) and *Klebsiella* sp. (1). Three patients did not present any bacterial growth. One of these patients with *Staphylococcus aureus* exhibited a secondary strain which was found to be *Citrobacter* sp. (1). These data demonstrate the common bacterial profiles found in SBE victims, and this is not hugely different for Russell’s viper bite victims.

### 2.3. Gender Does Not Correlate with the Growth of Diverse Bacterial Strains, but Age Does

To determine if gender can influence the type of bacteria growing in patients’ wounds, further analysis was performed. A chi-square test was used to examine the association between most bacterial strains present and gender. However, Fisher’s exact test was used where the sample numbers were small (*Enterococcus* sp. and *Proteus mirabilis*). The results (Table 1) demonstrate that there is no association between gender and type of bacterial strains found in SBE victims. However, the relationship between *Enterococcus* sp. (*p* = 0.05) and *Pseudomonas aeruginosa* (*p* = 0.08) and gender appeared to be close to significant.

We then analysed the association between age and bacterial strains. These results (Table 2) indicated that *Escherichia coli* (*p* = 0.02) and *Staphylococcus aureus* (*p* = 0.02) strains varied significantly between age groups. *Escherichia coli* was more commonly (27 out of 35 patients) observed in the age category of above 50 years old, whereas *Staphylococcus aureus* was commonly (37 out of 58 patients) observed in patients of below 50 years old. The other bacterial strains did not vary significantly between different age groups. These data demonstrate that there is an influence of age in specific types of bacteria growing in SBE victims, while gender may not be a concern.

### 2.4. Bacterial Sensitivity and Resistance to Antibiotics in Russell’s Viper Victims

**Figure 3:**
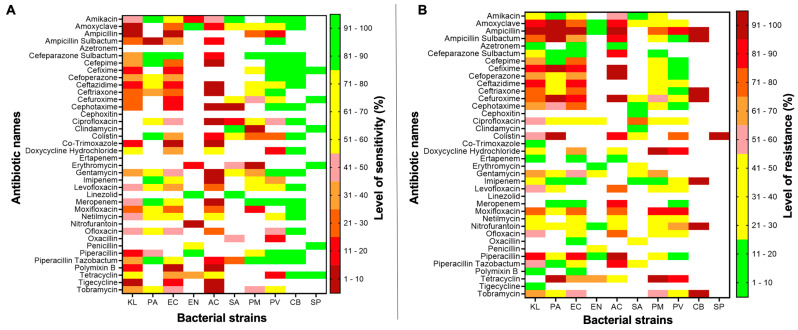
Antibiotic sensitivity data for different bacterial strains grown on bite sites of Russell’s viper bite victims. The sensitivity (**A**) and resistance (**B**) of diverse bacterial species grown at the bite sites of Russell’s viper victims for a broad range of antibiotics tested in this study. The scales shown in the figure indicate the level of sensitivity in (**A**) and resistance in (**B**) for antibiotics. KL—*Klebsiella* sp.; PA—*Pseudomonas aeruginosa*; EC—*Escherichia coli*; EN—*Enterococcus* sp.; AC—*Acinetobacter* sp.; SA—*Staphylococcus aureus*; PM—*Proteus mirabilis*; PV—*Proteus vulgaris*; CB—*Citrobacter*; SP—*Staphylococcus pyogenes*. White squares indicate the antibiotics that were not tested for the given bacterial strain.

Due to the large number of patients in Russell’s viper cohort compared to other snakes, we used only these data to analyse the sensitivity of bacterial strains to a range of antibiotics (Figure 3A). The data demonstrate that *Staphylococcus aureus* is most sensitive to linezolid (96%), amikacin (90%), clindamycin (84%), and colistin (66%). Similarly, the top four most effective antibiotics for the other most commonly identified bacterial strains such as *Klebsiella* sp. (doxycycline hydrochloride (62.5%), tetracyclin (59.4%), netilmycin (50%), and amikacin (50%)), *Pseudomonas aeruginosa* (colistin (100%), amikacin (91.7%), cefepime (87.5%), imipenem (87.5%), meropenem (87.5%), and cefeparazone sulbactum (83.3%)), and *Escherichia coli* (meropenem (78.6%), piperacillin tazobactum (75%), amikacin (67.9%) and netilmycin (64.3%)), were identified. The sensitivity of other bacterial strains to various antibiotics is shown in Figure 3A.

Similarly, the least effective antibiotics against bacterial strains grown in Russell’s viper bite victims were analysed (Figure 3B). The top four antibiotics that are resistant to *Staphylococcus aureus* include ciprofloxacin (76%), oxacillin (50%), gentamycin (46%), and amoxiclave (42%). *Klebsiella* sp. were resistant to ampicillin (97%), amoxiclave (84%), cefixime (84%), and piperacillin (81%). *Escherichia coli* were resistant to ampicillin (96%) piperacillin (86%), cefuroxime (86%), and ceftazidime (86%). Notably, *Pseudomonas aeruginosa* was fully resistant to several antibiotics such as amoxiclave (100%), ampicillin (100%), cefixime (100%), cefuroxime (100%), colistin (100%), tetracyclin (100%), and ampicillin sulbactum (95.8%). The resistance of other bacterial strains to various antibiotics is shown in Figure 3B.

### 2.5. The Cost Analysis of All Antibiotics Indicates Their Significance in SBE Treatment

**Figure 4:**
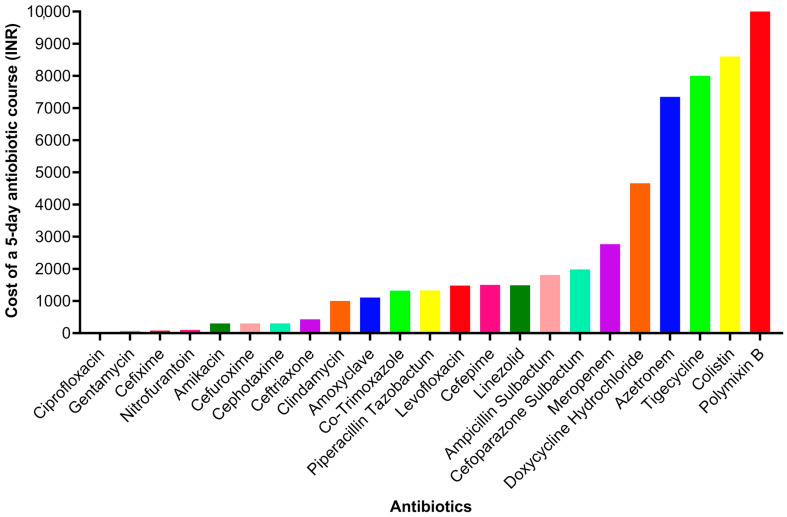
Cost of a 5-day antibiotic course (two doses daily) for different antibiotics that were used in SBE victims in this study.

The total costs of a 5-day course of antibiotics were calculated based on a twice-daily dosing (Figure 4). Three of the antibiotics (cefixime, cefuroxime, and ciprofloxacin) were provided as tablets, while all others were given as an intravenous infusion. The cheapest course of antibiotics was ciprofloxacin with the cost in Indian Rupees being INR 32. The most expensive antibiotics course was polymixin B, as it costs INR 10,000. The most effective antibiotic (linezolid) for *Staphylococcus aureus* costs INR 1490 for a 5-day course. Similarly, for *Klebsiella* sp., the effective antibiotic, doxycycline hydrochloride, costs around INR 4660. Colistin was identified as the most effective antibiotic against *Pseudomonas aeruginosa*, and it costs around INR 8600. For *Escherichia coli*, with the effective antibiotic, meropenem, treatment costs INR 2700. All the costs of different antibiotics are shown in Figure 4. The analysis of this cost data highlights that out of the ten bacterial strains identified, eight bacteria showed >80% sensitivity to at least one antibiotic tested. *Klebsiella* sp. and *Acinetobacter* sp. did not display >80% sensitivity to any antibiotic tested. Of the 38 antibiotics tested, 27 presented with at least 80% sensitivity to at least one bacterial strain. In this study, the patients received different antibiotics based on their level of sensitivity to the bacterial strain identified and the cost of antibiotics, which significantly varied (Figure 4).

## 3. Discussion

SBE is an acute medical emergency that can cause a range of different complications within the body [13]. Some of these complications are due to the direct actions of venom toxins and others develop as secondary complications to envenomation effects. Snake venoms (specifically from vipers such as the Russell’s viper) contain a combination of toxins that damage various cells and proteins, leading to blood clotting disorders, acute inflammation, and extensive damage to local tissues [14,15]. The local tissue damage creates an environment that is favourable for the growth of multiple pathogenic microorganisms, which can come from the environment, the skin, or even the oral cavity of the snake [11]. Indeed, the mouth of the Russell’s viper snake contains diverse types of bacteria, including antibiotic-resistant strains [12]. Additionally, the use of alternative and non-validated treatment methods such as plant extracts, cow dung, and other forms of traditional remedies in SBE-affected areas are common in rural areas [16]. These methods may also significantly increase the risk of developing microbial infections. Therefore, the clinical management of SBE should consider the risk of wound infections, including from antibiotic-resistant bacterial strains, and hence develop robust antibiotic strategies as part of the treatment protocol to successfully treat victims [17].

The use of antibiotics as a preventative measure following envenomation by snakes is controversial or unnecessary, and therefore, they should only be used when there is confirmation of a wound infection [18,19]. Overuse or unnecessary administration of antibiotics in SBE victims can contribute to antimicrobial resistance, undesirable side effects, and increased treatment costs [20,21]. Moreover, wound infections that are not treated quickly can lead to serious complications such as abscess, gangrene, and necrosis, which may require surgical intervention, adding a strain on already fragile healthcare systems with limited resources and poor infrastructure [22]. Hence, it is critical to improve our understanding of microbial/bacterial populations that grow in SBE-induced infections and develop robust strategies to tackle this issue. In this study, we analysed the types of bacteria and their sensitivity and resistance to a wide range of antibiotics using data collected from SBE victims who displayed visible wounds upon admission to a hospital. This study is aimed towards developing an empirical treatment regimen that can bring several potential benefits to SBE victims, including rapid response to severe infections, improving victim care in healthcare settings with limited resources, and avoiding the overuse or unnecessary use of ineffective antibiotics that can lead to resistance and increased treatment costs.

A total of 10 bacterial strains were identified in the wound swabs obtained from SBE victims in this study. *Staphylococcus aureus*, *Klebsiella* sp., *Pseudomonas aeruginosa*, and *Escherichia coli* were identified as the most common bacteria in SBE victims. These results are similar to previous reports describing the bacterial profiles of SBE-induced wounds in other countries and species of snakes [23,24]. For example, a study carried out in Taiwan evaluating the secondary infection in patients bitten by *Naja atra* identified a total of 23 bacterial strains [22]. Another study performed in Brazil described the presence of 54 different bacterial strains in abscesses developing at the bite site [25]. Some studies have shown the predominance of aerobic Gram-negative bacteria [22,25,26]. However, our results indicate the presence of certain specific Gram-positive and Gram-negative bacterial strains as the most commonly found bacteria in SBE victims in an Indian hospital. A previous study that analysed SBE victims over approximately 5 years (2003–2008) in an Indian hospital showed a higher prevalence of Gram-positive strains (53%) [27]. Garg et al. (2009) reported that *Staphylococcus aureus* (32%) was the most frequent microorganism followed by *Escherichia coli* (15%). Our data analysis highlighted the occurrence of *Staphylococcus aureus* in 31% of victims followed by a Gram-negative bacterium, *Escherichia coli*, in 17% of victims. Some of the bacteria identified in our study coincide with those previously reported in the oral cavity of Russell’s viper [12]. The mouth of Russell’s viper comprises a series of pathogenic bacteria that include *Staphylococcus* sp., *Enterococcus* sp., *Lysinobacillus* sp., *Escherichia coli*, *Pseudomonas* sp., *Salmonella* sp., *Proteus* sp., *Providencia* sp., *Morganella* sp., and *Alcaligenes* sp. However, the bacterial profiles or epidemiology of bacteria in wound infections have a different pattern of distribution to the bacteria found in the oral cavity of this snake. This suggests that some bacterial infections in SBE patients may be associated with the snake’s oral microflora but may not be all of them. Therefore, other sources of microbes, including the skin of the patient and any first aid or traditional treatment methods, were used post-snakebite. Moreover, the mouth of different snakes may have various microbial patterns which can influence the infection in patients. During this study period, there was no correlation found between the bacteria identified from the patient swabs and the nosocomial culture (no bacterial growth observed) that was obtained using swabs from theatres, intensive care units, and wards.

Antibiotic resistance is a pressing global issue that affects the wider clinical practice [28]. The inadequate efficacy and unnecessary use of antibiotics in treating SBE-induced infections can lead to serious consequences, including undesirable side effects [26]. Our study on antibiotic resistance and sensitivity to a wide range of bacteria through a robust assessment using a large cohort of patients provides practical guidelines for selecting effective antibiotics to treat SBE-induced infections in victims. Our results showed that antibiotics such as linezolid, amikacin, clindamycin, piperacillin tazobactum, cefeparazone sulbactum, and colistin are some of the most effective to treat a range of bacteria identified in the wound swabs of SBE victims. Similarly, ampicillin, cefuroxime, cefixime, and amplicillin sulbactum are some of the least effective antibiotics for bacterial strains identified. This is in accordance with a previous study [12] that analysed the susceptibility of bacteria from Russell’s viper’s mouth as it includes ampicillin, cefpodoxime, amoxiclave, oxacillin, and penicillin as resistant antibiotics. These data provide a comprehensive profile of sensitivity and resistance to a range of antibiotics based on the type of bacteria. These data can be used as a potential guide to determine the antibiotic regime for SBE victims.

Vulnerable populations with scarce economic resources face high treatment costs for SBE, which can be largely impacted by secondary effects [1]. A retrospective analysis performed in Kenya has highlighted that SBE-related complications have significant consequences on medical/pharmacy costs and supportive therapies that are key determinants for the total high treatment costs [29]. However, the number of investigations focused on the estimations of the cost of SBE treatments is very limited. The economic impact of antibiotic therapy remains widely unknown [2,29,30]. Here, we showed that the treatment costs can vary drastically depending on the antibiotics used. These findings draw attention to the necessity of designing effective and affordable antibiotic treatment strategies that can reduce the cost and, consequently, the burden that this issue represents for the population in low-income regions. More robust studies are required to tackle this issue across the world and develop appropriate treatment guidelines and policies.

## 4. Conclusions

This study highlights the importance of understanding the bacterial community in wound infections from SBE victims, specifically Russell’s viper bite victims. Moreover, antibiotic sensitivity tests were performed to improve treatment plans and clinical guidelines. In rural areas with limited resources, identifying microbes in SBE victims can be challenging and time-consuming, hindering prompt treatment. Therefore, a systematic review of microbial data from low-resource settings is necessary to establish efficient management protocols. However, this study has limitations due to its size, despite having the most data from one of the most medically significant snake species (Russell’s viper) in the country. Indeed, the development of visible wounds from other snakebite victims was minimal. Therefore, further studies are needed to fully understand the bacterial communities in the remaining three deadliest Indian snakes. This study used a robust automated culture technique for rapid bacterial identification, and this may not be available in rural healthcare settings. Moreover, some studies that used mass spectrometry-based methods have been proposed as faster and more sensitive alternatives for diagnosing bacterial infections, and should be considered in future studies. In conclusion, a better understanding of microbial/bacterial communities that are responsible for SBE-induced infections and their sensitivity/resistance to a range of antibiotics will significantly improve the treatment and reduce treatment costs for SBE victims.

## 5. Materials and Methods

### 5.1. Data Collection

This prospective study was conducted from January 2021 to December 2022 at Manian Medical Centre (a snakebite referral hospital), Erode, Tamil Nadu, India. All patients of any age who displayed a visible wound or bleeding upon admission were included in this study. The patients bitten by only the ‘Big Four’ snakes were included, and others were excluded. The identity of the snakes was confirmed based on dead/live specimens brought by the victims or their family members, and/or clinical symptoms displayed. A trained herpetologist has analysed the identification of snakes where specimens were available based on their morphological features. A total of 266 patients were included in this study. Due to the low number of cases of snakes other than Russell’s viper, the data obtained from Russell’s viper bite victims (242 cases) was used for most of the analysis in this study to draw firm conclusions.

Sterile cotton microbiological swabs (HiMedia, India) were used to collect the wound exudates at the bite site of patients and immediately sent [in HiCulture^TM^ swab transport media containing Dey-Engley neutralising broth, (HiMedia, India)] for microbial culture in the laboratory associated with the hospital. The swabs were streaked using the quadrant method on blood and Macconkey agar media plates. They were incubated at 37 °C for 48 h for the bacteria to grow. If there was no bacterial growth observed at this stage, the plates were monitored for another 48 h with an examination every 12 h. Then, single well-isolated colonies of bacteria were selected for further microbial characterisation using a Gram stain kit (HiMedia, India) and a range of biochemical tests [e.g., Indole, citrate, methyl red, oxidase, triple sugar iron, and coagulase tests using HiIMViC^TM^ Biochemical test kits] according to the manufacturer’s guidelines (HiMedia, India).

Similarly, the isolated colonies were grown in separate Muller Hinton agar plates and used for antibiotic sensitivity tests using a range of antibiotic discs in line with the manufacturer’s (HiMedia, India) guidelines. Briefly, the susceptibility test measures the zone of clearance for each antibiotic disc following the guidelines from the Clinical & Laboratory Standards Institute (CLSI) and the European Committee on Antimicrobial Susceptibility Testing (EUCAST). The zone of clearance or antibiotic susceptibility was measured using an automatic analyser (Vitek 2 compact analyser, Biomerieux, India). Similarly, this analyser has calculated the minimum inhibitory concentrations (MIC) for susceptible antibiotics based on the zone of clearance observed. If there was no zone of clearance, then those bacteria were considered resistant to that antibiotic. To determine the nosocomial infections, the swabs from operation theatres, intensive care units, and wards were routinely tested for every 15 days. There was no bacterial growth identified from these swabs as the hospital is maintaining stringent surface cleaning protocols.

### 5.2. Statistical Methods

All statistical analyses were performed using SPSS version 26 (IBM, Portsmouth, UK) and R version 4.1.2 (Lucent technologies Ltd, Manchester, UK) to evaluate the association between the age category and gender of the patients and the presence of various strains of bacteria. Due to the relatively small patient sample within each age group, the analysis for age was performed using Fisher’s exact test. A chi-square test was used to study the association with the bacterial strain presence.

## Figures and Tables

**Table 1:** Association between gender and occurrence of different bacterial strains.

Strain *	Female (*n* = 63)	Male (*n* = 126)	*p*-Value
AC	4 (6%)	9 (7%)	0.84
EC	12 (19%)	20 (16%)	0.58
EN	2 (3%)	15 (12%)	0.05
KL	10 (16%)	26 (21%)	0.43
PA	13 (21%)	14 (11%)	0.08
PM	3 (5%)	14 (11%)	0.19
SA	22 (34%)	36 (29%)	0.37

* AC—*Acinetobacter* sp., EC—*Escherichia* coli, EN—*Enterococcus* sp., KL—*Klebsiella* sp., PA—*Pseudomonas aeruginosa*, PM—*Proteus mirabilis* and SA—*Staphylococcus aureus*.

**Table 2:** Association between age and occurrence of diverse bacterial strains.

Strain *	Age ≤ 20 (*n* = 10)	Age 21–30 (*n* = 12)	Age 31–40 (*n* = 22)	Age 41–50 (*n* = 44)	Age 51–60 (*n* = 48)	Age 61–70 (*n* = 33)	Age 71+ (*n* = 20)	*p*-Value
AC	1 (10%)	0 (0%)	2 (9%)	2 (5%)	5 (10%)	2 (6%)	1 (5%)	0.88
EC	0 (0%)	1 (8%)	1 (5%)	6 (7%)	13 (27%)	9 (27%)	5 (25%)	0.02
EN	0 (0%)	1 (8%)	1 (5%)	6 (14%)	3 (6%)	4 (12%)	2 (10%)	0.74
KL	2 (20%)	3 (25%)	9 (41%)	5 (12%)	7 (15%)	5 (15%)	2 (25%)	0.12
PA	2 (20%)	2 (17%)	3 (14%)	3 (14%)	7 (15%)	8 (24%)	2 (10%)	0.51
PM	0 (0%)	0 (0%)	0 (0%)	9 (21%)	4 (8%)	2 (6%)	2 (10%)	0.12
SA	5 (50%)	5 (42%)	6 (27%)	21 (48%)	13 (27%)	4 (12%)	4 (20%)	0.02

* AC—*Acinetobacter* sp., EC—*Escherichia coli*, EN—*Enterococcus* sp., KL—*Klebsiella* sp., PA—*Pseudomonas aeruginosa*, PM—*Proteus mirabilis* and SA—*Staphylococcus aureus*. The *p* values shown in bold denote the statistical significance for the association of those bacterial strains with the age of victims.

## Data Availability

Not applicable—as all data from this study are included within this article.
